# Cannabis Use in Adults Who Screen Positive for Attention Deficit/Hyperactivity Disorder: CANreduce 2.0 Randomized Controlled Trial Subgroup Analysis

**DOI:** 10.2196/30138

**Published:** 2022-04-20

**Authors:** Joachim Ahlers, Christian Baumgartner, Mareike Augsburger, Andreas Wenger, Doris Malischnig, Nikolaos Boumparis, Thomas Berger, Lars Stark, David D Ebert, Severin Haug, Michael P Schaub

**Affiliations:** 1 Swiss Research Institute for Public Health and Addiction University of Zurich Zurich Switzerland; 2 Institute for Addiction Prevention Office of Addiction and Drug Policy of Vienna Vienna Austria; 3 Department of Clinical Psychology and Psychotherapy University of Bern Bern Switzerland; 4 Arud Centre for Addiction Medicine Zurich Switzerland; 5 Clinical Department for Sport and Health Sciences Technical University Munich Munich Germany

**Keywords:** attention deficit/hyperactivity disorder, ADHD, cannabis, cannabis use disorder, CANreduce, web-based self-help tool, online tool, online health, mental health, digital health, anxiety, depression

## Abstract

**Background:**

Prevalence rates for lifetime cannabis use and cannabis use disorder are much higher in people with attention deficit/hyperactivity disorder than in those without. CANreduce 2.0 is an intervention that is generally effective at reducing cannabis use in cannabis misusers. This self-guided web-based intervention (6-week duration) consists of modules grounded in motivational interviewing and cognitive behavioral therapy.

**Objective:**

We aimed to evaluate whether the CANreduce 2.0 intervention affects cannabis use patterns and symptom severity in adults who screen positive for attention deficit/hyperactivity disorder more than in those who do not.

**Methods:**

We performed a secondary analysis of data from a previous study with the inclusion criterion of cannabis use at least once weekly over the last 30 days. Adults with and without attention deficit/hyperactivity disorder (based on the Adult Attention deficit/hyperactivity disorder Self-Report screener) who were enrolled to the active intervention arms of CANreduce 2.0 were compared regarding the number of days cannabis was used in the preceding 30 days, the cannabis use disorder identification test score (CUDIT) and the severity of dependence scale score (SDS) at baseline and the 3-month follow-up. Secondary outcomes were Generalized Anxiety Disorder score, Center for Epidemiological Studies Depression scale score, retention, intervention adherence, and safety.

**Results:**

Both adults with (n=94) and without (n=273) positive attention-deficit/hyperactivity disorder screening reported significantly reduced frequency (reduction in consumption days: with: mean 11.53, SD 9.28, *P*<.001; without: mean 8.53, SD 9.4, *P*<.001) and severity of cannabis use (SDS: with: mean 3.57, SD 3.65, *P*<.001; without: mean 2.47, SD 3.39, *P*<.001; CUDIT: with: mean 6.38, SD 5.96, *P*<.001; without: mean 5.33, SD 6.05, *P*<.001), as well as anxiety (with: mean 4.31, SD 4.71, *P*<.001; without: mean 1.84, SD 4.22, *P*<.001) and depression (with: mean 10.25, SD 10.54; without: mean 4.39, SD 10.22, *P*<.001). Those who screened positive for attention deficit/hyperactivity disorder also reported significantly decreased attention deficit/hyperactivity disorder scores (mean 4.65, SD 4.44, *P*<.001). There were no significant differences in change in use (*P*=.08), dependence (*P*=.95), use disorder (*P*=.85), attention deficit/hyperactivity disorder status (*P*=.84), depression (*P*=.84), or anxiety (*P*=.26) between baseline and final follow-up, dependent on positive attention-deficit/hyperactivity disorder screening. Attention deficit/hyperactivity disorder symptom severity at baseline was not associated with reduced cannabis use frequency or severity but was linked to greater reductions in depression (Spearman *ρ*=.33) and anxiety (Spearman *ρ*=.28). Individuals with positive attention deficit/hyperactivity disorder screening were significantly less likely to fill out the consumption diary (*P*=.02), but the association between continuous attention deficit/hyperactivity disorder symptom severity and retention (Spearman *ρ*=−0.10, *P*=.13) was nonsignificant. There also was no significant intergroup difference in the number of completed modules (with: mean 2.10, SD 2.33; without: mean 2.36, SD 2.36, *P*=.34), and there was no association with attention deficit/hyperactivity disorder symptom severity (Spearman *ρ*=−0.09; *P*=.43). The same was true for the rate of adverse effects (*P*=.33).

**Conclusions:**

Cannabis users screening positive for attention deficit/hyperactivity disorder may benefit from CANreduce 2.0 to decrease the frequency and severity of cannabis dependence and attenuate symptoms of depression and attention deficit/hyperactivity disorder-related symptoms. This web-based program’s advantages include its accessibility for remote users and a personalized counselling option that may contribute to increased adherence and motivation to change among program users.

**Trial Registration:**

International Standard Randomized Controlled Trial Number (ISRCTN) 11086185; http://www.isrctn.com/ISRCTN11086185

## Introduction

The worldwide prevalence of attention deficit/hyperactivity disorder is estimated to be 5% in children and up to 4% in adults [1-3]. Lifetime prevalence rates for cannabis use are rising [4], with current rates of 26% in Europe [5], 46% in the United States [6], and 28% in Switzerland (4). Lifetime cannabis use is significantly more common in people with attention deficit/hyperactivity disorder (66.1%) than in those without (46.9%); similarly, cannabis use disorders are significantly more common in people with attention deficit/hyperactivity disorder (23.5%) than in those without (8%) [7].

Cannabis is predominantly seen as a safe drug in Western societies [8]. Life satisfaction and stress do not seem to predict the initiation, cessation, or severity of cannabis use in young adults [9,10]; however, research indicates a higher prevalence of various mental health symptoms in cannabis users, such as depression, anxiety [10-13], and attention deficit/hyperactivity disorder, with increased severity in certain populations such as those in the Czech Republic and France [14-16].

In addition, meta-analyses have documented that attention deficit/hyperactivity disorder alone is linked to high comorbidity rates of mental health symptoms, such as anxiety disorders [17] and depression [18], across all life stages.

Recent research [19] showed a causal genetic link between attention deficit/hyperactivity disorder and lifetime cannabis use and emphasizes the hereditary nature of both entities. Specifically, the heritability of attention deficit/hyperactivity disorder is estimated at 70%-80% and of cannabis use initiation at 40%-48% [19].

Cognitive behavioral therapy is an effective treatment option for adults with attention deficit/hyperactivity disorder [20]. Furthermore, web-based cognitive behavioral therapy has been shown to be more effective for cannabis users with attention deficit/hyperactivity disorder than for those without [21]. In addition, the first study [22] that assessed a web-based intervention for people with attention deficit/hyperactivity disorder found it to be successful alleviating attention deficit/hyperactivity disorder symptoms.

There is evidence that integrated cognitive behavioral therapy—a combination of 2 research-based cognitive behavioral therapy methods (one for substance use disorder and one for attention deficit/hyperactivity disorder)—performs significantly better than regular addiction treatment, such as cognitive behavioral therapy alone, among attention deficit/hyperactivity disorder patients with cannabis use disorder. Patel et al [23] found that, in adolescents with attention deficit/hyperactivity disorder, comorbid cannabis use disorder is associated with a 90% lower likelihood of successful attention deficit/hyperactivity disorder treatment outcomes than that without comorbid cannabis use disorder. A recent randomized controlled trial revealed that, compared to regular substance use disorder cognitive behavioral therapy, integrated cognitive behavioral therapy resulted in significantly greater improvement in attention deficit/hyperactivity disorder symptoms in patients with substance use disorder and attention deficit/hyperactivity disorder [24].

Given the high comorbidity rates of attention deficit/hyperactivity disorder and substance use disorder, findings with respect to individuals with attention deficit/hyperactivity disorder and substance use disorder are promising and should be further evaluated to improve treatment outcomes for individuals with attention deficit/hyperactivity disorder and comorbid cannabis use disorder.

Among studies assessing web-based treatments designed to reduce cannabis use, none has considered whether adults with attention deficit/hyperactivity disorder were included or whether attention deficit/hyperactivity disorder is a potential moderator of treatment effectiveness.

Web-based interventions are known for removing barriers against seeking help for addictions, particularly stigmatization and inadequate access to treatment facilities [25]. Even though there is a lack of comparable studies using web-based programs to guide cannabis users with attention deficit/hyperactivity disorder through their process of reducing or quitting substance use, the effect of cannabis use on persons with attention deficit/hyperactivity disorder and the characteristics of this subgroup of cannabis users have been investigated. Soler Artigas et al [19] discovered a causal relationship between attention deficit/hyperactivity disorder and cannabis use, based on the identification of specific loci predisposing individuals to these traits, which showed that patients with attention deficit/hyperactivity disorder have a 7.9-fold increase in the odds of using cannabis than persons without this condition. Wallace et al [26] revealed that the symptoms associated with attention deficits—such as low scores on neuropsychological performance tests—were likely attributable to cannabis use itself rather than to attention deficit/hyperactivity disorder. Brandt et al [27] retrospectively investigated clinical parameters that correlated with cannabis use among respondents with and without attention deficit/hyperactivity disorder in the National Epidemiologic Survey on Alcohol and Related Conditions, and although 14.3% of the respondents with attention deficit/hyperactivity disorder used cannabis, only 4.3% of those without attention deficit/hyperactivity disorder used cannabis. There was also a significantly higher prevalence of psychiatric and personality disorders in respondents with attention deficit/hyperactivity disorder who consumed cannabis than in those who did not [27]. Patel et al [23] retrospectively evaluated patient data from adolescents with attention deficit/hyperactivity disorder and comorbid cannabis use disorder and observed this patient group’s increased need for acute care. A similar study [28] revealed that a perception exists, among attention deficit/hyperactivity disorder–patient web-based forum participants, that cannabis is beneficial and reduces symptoms associated with the condition.

Several recent meta-analyses [25,29-31] have demonstrated the overall effectiveness and great potential of web-based prevention and treatment interventions for cannabis use reduction but that such studies have typically been plagued by high dropout rates. Thus, further analysis of web-based interventions seeking to reduce cannabis use in adults with comorbid attention deficit/hyperactivity disorder is warranted.

In a recent 3-arm randomized controlled trial (CANreduce 2.0 study) [32], we examined the effects of an enhanced self-guided web-based intervention tool with a social presence in treatment-seeking adults who overuse cannabis. The social presence included an eCoach for supportive accountability and human support to enhance adherence to the eHealth intervention. We found moderate to medium effects in the reduction of cannabis use days and significant effects influencing secondary cannabis related outcomes, as well as reducing general anxiety disorder symptoms, when compared to an internet-as-usual control group [33].

Previously published studies [27,34-36] have demonstrated that individuals with attention deficit/hyperactivity disorder may be particularly at risk of using cannabis, that the severity of cannabis involvement is significantly associated with greater endorsement of attention deficit/hyperactivity disorder symptoms [34], and that diagnosis of any psychiatric disorder is significantly higher among those with attention deficit/hyperactivity disorder and concurrent cannabis use [27]. Furthermore, cannabis use has been shown to interact with and decrease the beneficial effects of commonly prescribed medication for individuals with attention deficit/hyperactivity disorder [35]. In addition, cannabis use severity seems to worsen attention deficit/hyperactivity disorder symptoms [36]. Therefore, we anticipated that individuals screening positive for attention deficit/hyperactivity disorder might particularly benefit from the CANreduce 2.0 intervention compared to individuals without attention deficit/hyperactivity disorder symptoms.

The aim of this study was to gain insights into the impact of attention deficit/hyperactivity disorder symptoms on outcomes when individuals who overuse cannabis participate in a web-based intervention, in terms of the program’s efficacy reducing cannabis use, while also examining how severity is affected. In particular, we sought to answer the following question: Does attention deficit/hyperactivity disorder severity correlate with change in cannabis consumption posttreatment? Secondary outcomes were changes in attention deficit/hyperactivity disorder symptom severity, intervention adherence and retention, and how safe the intervention was perceived to be. We hypothesized (1) that the CANreduce 2.0 intervention would reduce cannabis use and associated mental health problems (ie, depression and anxiety) in participants whether they screened positive or negative for attention deficit/hyperactivity disorder; (2) that participants screening positive for attention deficit/hyperactivity disorder would benefit to a greater extent, in terms of cannabis use reduction, than those screening negative for attention deficit/hyperactivity disorder; and (3) that baseline attention deficit/hyperactivity disorder symptom severity would correlate with intervention-related changes in other outcomes.

## Methods

### Study Design

We conducted secondary analysis of the CANreduce 2.0 data set [33]. The sample was extracted from the 2 active intervention arms (n=367), excluding all individuals in the internet-as-usual group because the study’s purpose was not to determine whether either active intervention was effective relative to a control condition but to compare the degree of effect in patients screening positive versus screening negative for attention deficit/hyperactivity disorder. These 367 adults included 94 young adults who screened positive and 273 who screened negative for comorbid attention deficit/hyperactivity disorder. For our analysis, participants with attention deficit/hyperactivity disorder in the 2 active intervention arms were pooled, as were those who screened negative.

In the original 3-arm RCT, 2 active arms with an adherence-focused, guidance enhanced, web-based self-help intervention with and without a mostly automated personal eCoach were compared with a nonactive arm (waiting-list controls with access to internet services as usual). The concept of adherence-focused guidance stems from observations that guided self-help programs are more effective than programs without guidance, based on the supportive-accountability model [37]. Each of the 2 active interventions consisted of 8 modules specifically developed to decrease cannabis use and reduce symptoms of common mental disorders such as attention deficit/hyperactivity disorder, anxiety, and depression. Module content was based on the strategies of motivational interviewing and cognitive behavioral therapy. Study participants were assessed after the intervention, by comparing their baseline characteristics with reports obtained at the 3-month follow-up assessment. Retention of participants in these subgroups was evaluated weekly until the end of the intervention.

### Recruitment

Recruitment took place from August 2016 through December 2018. Potential participants were recruited via the CANreduce websites [38,39] and associated health-related websites linked to the study. Recruitment also was achieved through advertisements in relevant internet forums and newspapers (or web-based versions thereof) and search engine website advertisements. The recruitment process was not attention deficit/hyperactivity disorder–specific—recruitment was neither addressed to persons with attention deficit/hyperactivity disorder, nor performed in attention deficit/hyperactivity disorder-related institutions, websites, or forums. After completing the 3-month follow-up survey, participants were offered either a voucher (30€, approximately US $33.50) or the choice to donate the equivalent amount to charity.

### Consent Procedure, Registration, and Randomization

Participants could register on the website and had to provide only minimal personal data (email address; a phone number to be contacted, if follow-up questionnaires were not filled out; and basic demographic data), in accordance with the CANreduce 2.0 research protocol.

### Inclusion and Exclusion Criteria

Interested individuals initially were informed about the purpose, background, and structure of the study. They were provided with information on the inclusion and exclusion criteria (Table 1), followed by information on ethical safe-guards (the right to withdraw at any time, confidentiality) and on data protection and safety arrangements. Informed consent consisted of activating several check boxes that restated important study points and clicking a consent submission button. Potential participants who stated they were in any psychosocial or psychiatric treatment for their cannabis use disorder were excluded. There was no question exploring previous face-to-face attention deficit/hyperactivity disorder diagnostic or related medication, since the rate of participants receiving current psychiatric or pharmacological treatment for adult attention deficit/hyperactivity disorder was expected to be low. Those still interested and eligible were then asked to register on the website and complete a baseline assessment, after which they were randomized by a computer algorithm in a 1:1:1 ratio into 1 of the 3 study arms (2 active, 1 control). Participants in study arms 1 and 2 were introduced, step by step, to their intervention, while those in study arm 3 were informed that they would be granted access to the intervention after they completed their follow-up assessment 3 months later.

**Table 1:** Inclusion and exclusion criteria with rationales.

Criterion			Rationale
**Inclusion**			
	Informed consent via a web form	Ensure knowledge of procedures and declaration of consent	
	At least 18 years old	Ensure minimum age of participation	
	Cannabis use at least once weekly over the last 30 days	Include participants with less than daily cannabis use and increase validity	
	Internet access at least once weekly and a valid email address	Ensure access to the intervention	
	Good command of the German language	Ensure that participants understand the information provided	
**Exclusion**			
	Participation in other psychosocial or pharmacological treatments for the reduction or cessation of cannabis use	Avoid confounding treatment effects	
	Current pharmacologically treated psychiatric disease or any history of psychosis, schizophrenia, bipolar type I disorder, or significant current suicidal or homicidal thoughts	Prevent individuals with such problems from entering the study	

### Study Interventions

CANreduce version 1.0 [40,41] had already been shown to be effective at reducing cannabis use by combining an automated self-help program (web-based psychoeducation modules with a consumption diary) with the opportunity for individual chat counseling, both of which were grounded in motivational interviewing, self-control practices, and classical cognitive behavioral therapy. However, there were difficulties with adherence, retention, and high dropout rates.

The current version (CANreduce 2.0) was designed to overcome these difficulties, with the implementation of additional adherence-focused guidance—feedback on demand and enhanced adherence monitoring mainly through motivational automated emails with weekly reminders, encouragement, and suggestions for further self-help module interventions, and the constant opportunity for participants to ask any questions they might have throughout their participation in the web-based program.

The 2 active study arm groups received the same level of enhanced support, but only study arm 1 received specific enhancements with a social presence, based on Mohr’s supportive accountability model [37]. This involved more intimate, personally addressed emails, texts, and videos from a constantly visualized eCoach, with the intention of creating a more personalized atmosphere, greater alliance between the user and eCoach, and a more considerate and caring participant–counselor connection, and with no explicit need for a certified therapist as constant backup.

### Modules, Dashboard, and Consumption Diary

The CANreduce 2.0 self-help intervention consists of 8 modules that encompass motivational interviewing techniques, traditional cognitive behavioral therapy, self-control practices, and social problem-solving [32]. Modules 1 and 2 are an introduction to the program and its application, helping program users to work through and identify individual triggers and triggering situations, so they can avoid unintentional cannabis use (motivational interviewing techniques [42], cognitive behavioral therapy approach to relapse prevention [43]). In Modules 3-5, skills and techniques are taught to enhance social relationships, restore sleeping patterns, deal with ruminations (behavioral activation approach [44]), and overcome situations considered risky for relapse, such as feelings of discouragement (cognitive behavioral therapy approach for relapse prevention [43] and to handle cravings [45]). By establishing rules and rituals and applying mindful positive thinking, self-talk, envisioning consequences, and distraction techniques, the program’s aim is for users to persevere in their attempts to reduce their cannabis consumption. Modules 6-8 teach problem solving (social problem-solving approach [46]) and rejection skills to help users resist cannabis use that exceed their individual cannabis consumption plan (based on cognitive behavioral therapy [45]); to meet challenges in daily life, such as manifestations of depression; and, upon program completion, to provide them with the opportunity for a personalized review (motivational interviewing techniques [43]).

The advanced version of CANreduce (2.0) is particularly tailored to patients with a common mental disorder such as attention deficit/hyperactivity disorder, to enhance program adherence and achieve better outcomes via improved module content that focuses on dis–related problems. These include associated common mental disorder symptoms such as depressed mood, and problem-solving skills (Module 6), as well as excessive ruminations and poor sleeping habits (Module 3). In addition, to help participants with attention deficit/hyperactivity disorder who are known to be easily distracted, coping strategies (such as focusing on cravings and letting them come and go) are evaluated (Module 4).

The concept of adherence-focused guidance was implemented to help participants who screened positive for attention deficit/hyperactivity disorder focus on the tasks in the modules. As an element of adherence monitoring, automated emails for motivation and emails for information on feedback on demand were sent to the participants.

The web-based program has a dashboard as its starting point that includes an overview of all 8 modules, providing useful information on a chronological timeline with follow-ups and data acquisition. Program users are advised to fill in their consumption diary at least once weekly, wherein participants in both active study arms were asked to define their individual standardized joint (on the basis of 36 photographs with specific cannabis doses) and their cannabis reduction goals at baseline. Participants could choose from 6 different fictional companions to accompany them through the program by communicating written thoughts and questions for further encouragement and reflection.

### Ethics and Data Protection

The study was conducted in accordance with the Declaration of Helsinki, the European Directive on medical devices 93/42/EEC, and the ISO Norm 14155 and ISO 14971 of Swiss Law and Swiss Regulatory Authority requirements [32]. The study was approved by the ethics committee of the Canton of Zurich (2016-00264) and is registered (ISRCTN11086185).

### Outcome Measures and Instruments

Sociodemographic data that were obtained included sex, age, country of origin, and highest level of education. Baseline characteristics pertaining to substance use included the number of cannabis joints consumed over the preceding 7 days, years of cannabis use, age at which regular cannabis use started, years of cannabis use, and any other substances consumed over the 30 days immediately preceding the study.

The study’s primary outcome was change in the number of days in which cannabis was consumed over the preceding 30 days according to the timeline follow-back method [47], which was compared between baseline and the 3-month follow-up assessment. Secondary outcomes were changes in the Severity of Dependence Scale (SDS) score, which can range from 0 to 15, with a score >4 indicating cannabis dependence [48]; cannabis use disorder identification test score (CUDIT), which can range from 0 to 40, with a score ≥ 8 indicating hazardous cannabis use and a score ≥12 indicating possible cannabis use disorder [49]; symptoms (cut off score >14) reported using the ADHD Self-Report scale (ASRS) version 1.1, which can range from 0 to 24 [3]; depression cut off score >16, using Center for Epidemiologic Studies Depression (CES-D), which can range from 0 to 60 [50]; anxiety, using the Generalized Anxiety Disorder scale (GAD) cut off score >10, which can range from 0 to 21 [51]; and Posttraumatic Stress Disorder short screening scale score, which can range from 7 to 28, with a cutoff sum score ≥4 suggesting posttraumatic stress disorder. Additional outcomes of interest were participant retention throughout the course of the study (defined as each person’s weekly diary completion rate), level of adherence to the intervention (defined as the number of modules each person completed), and any perceived adverse effects that participants attributed to the program. Study outcomes are described in greater detail elsewhere [31].

### Statistical Analysis

Sociodemographic parameters and baseline clinical characteristics were compared between those screening positive and negative for attention deficit/hyperactivity disorder, using Pearson chi-square analysis for categorical variables, analysis of variance for continuous variables, and the Kruskal-Wallis H test for ordinal variables.

Main outcomes of interest were compared between baseline and 3-month follow-up using paired *t* tests within each group—individuals with attention deficit/hyperactivity disorder (screened positive) and individuals without attention deficit/hyperactivity disorder (screened negative). An ASRS score >13 was considered attention deficit/hyperactivity disorder positive. Linear regression analyses were conducted with attention deficit/hyperactivity disorder screening group allocation as the predictor variable, controlled for the baseline value of the respective outcome variable to compare the individuals with attention deficit/hyperactivity disorder and individuals without attention deficit/hyperactivity disorder screen groups. To identify associations between attention deficit/hyperactivity disorder symptom severity and reductions in the primary and secondary outcomes after the intervention, Spearman rank correlation coefficients were calculated. The Fisher exact (2-tailed) test was used to compare the occurrence of adverse effects between individuals who screened positive and individuals who screened negative for attention deficit/hyperactivity disorder. Rates of retention, adherence, and perceived adverse effects were compared between individuals with attention deficit/hyperactivity disorder and individuals without attention deficit/hyperactivity disorder screen using 2-tailed paired *t* tests. We used intention-to-treat analysis. Missing values were imputed by means of chained equations with 20 sets of imputations [32]. The criterion for statistical significance was *P*<.05, and all inferential testing was 2-tailed.

## Results

### Baselines Characteristics of the Study Participants

Participants were predominantly male (263/367, 71.6%). The mean age was 27.9 years old (SD 7.5 years). No significant differences in age (*P*=.15) or the highest level of education (*P*=.36) between individuals with and without attention deficit/hyperactivity disorder were detected. The largest percentage of participants were from Switzerland (140/367, 38.1%), followed by Austria (134/367; 36.5%) and Germany (91/367, 24.7%).

All participants screened higher than the cutoff value (CUDIT ≥8) for cannabis use disorder. In the group with attention deficit/hyperactivity disorder, both the CUDIT and the SDS score were significantly higher (*P*<.001) than those in individuals screening negative. Scale scores for common mental disorder were also significantly higher (anxiety: *P*<.001; depression: *P*<.001; posttraumatic stress disorder: *P*=.005) in individuals with attention deficit/hyperactivity disorder than in those without.

The mean age of participants when they first started using cannabis was 20.0 years (SD 5.3 years), with a mean 7.9 years (SD 6.7 years) since the start of cannabis use. There were no statistically significant differences between groups for either starting age (*P*=.45) or duration of use (*P*=.31). Over the 7 days preceding the CANreduce 2.0 intervention, participants smoked a mean of 21.9 standard joints (SD 15.8 joints) per week. All participants reported cannabis consumption within the preceding 30 days, and 37% (134/367) reported risky alcohol use, which was defined as the consumption of 5 or more standard drinks on a single occasion (Table 2).

**Table 2:** Baseline characteristics of the study participants, by group and overall.

Characteristic			With attention deficit/hyperactivity disorder (n=94)		Without attention deficit/hyperactivity disorder (n=273)		All (n=367)		*F* test (*df1*,*df2*) or chi-square (*df*)^a^		*P* value
**Sex, n (%)**									0.58 (1)^a^		.45
	Female	30 (31.9)		74 (27.1)		104 (28.3)					
	Male	64 (68.0)		199 (72.8)		263 (71.6)					
Age, mean (SD)			26.9 (7.5)		28.2 (7.5)		27.9 (7.5)		2.10 (1,365)		.15
**Highest education level, n (%)**									13.16 (5)^a^		.36
	Compulsory school	10 (10.6)		16 (5.8)		26 (7.0)					
	Apprenticeship	17 (18.0)		46 (16.8)		63 (17.1)					
	Middle school	35 (37.2)		79 (28.9)		114 (31.0)					
	Higher professional education	11 (11.7)		44 (16.1)		55 (14.9)					
	University	17 (18.0)		76 (27.8)		93 (25.3)					
	Not stated	4 (4.2)		12 (4.3)		16 (4.3)					
**Origin, n (%)**									0.82 (2)^a^		.66
	Switzerland	39 (41.4)		101 (36.9)		140 (38.1)					
	Germany	24 (25.5)		67 (24.5)		91 (24.7)					
	Austria	31 (32.9)		103 (37.7)		134 (36.5)					
	Unknown	0 (0)		2 (0.7)		2 (0.5)					
Number of cannabis joints in preceding 7 days, mean (SD)			24.0 (14.4)		21.2 (16.2)		21.9 (15.8)		2.16 (1,365)		.14
Number of days cannabis (≥1 joint) was consumed in preceding 30 days, mean (SD)			26.6 (4.9		25.0 (6.4)		25.4 (6.1)		4.76 (1,365)		.03
Duration cannabis use (in years), mean (SD)			7.3 (6.1)		8.1 (6.9)		7.9 (6.7)		1.03(1,365)		.31
Age of onset of regular cannabis use (in years), mean (SD)			19.6 (5.5)		20.1 (5.2)		20.0 (5.3)		0.58 (1,365)		.45
Cannabis Use Disorder Identification Test score, mean (SD)			23.7 (4.6)		19.6 (5.6)		20.6 (5.7)		41.10 (1,365)		<.001
Adult ADHD Self-Report scale score, mean (SD)			15.8 (1.9)		9.0 (2.9)		10.7 (4.0)		438.60 (1,365)		<.001
Severity of Dependence Scale score, mean (SD)			9.1 (3.0)		7.1 (3.1)		7.6 (3.2)		31.01 (1,365)		<.001
Center for Epidemiological Studies Depression scale score, mean (SD)			27.4 (9.1)		19.8 (10.5)		21.7 (10.7)		39.40 (1,365)		<.001
Generalized Anxiety Disorder scale score, mean (SD)			10.7 (4.9)		6.6 (4.5)		7.6 (4.9)		55.90 (1,364)		<.001
Posttraumatic Stress Disorder Short Screening scale score, mean (SD)			14.9 (4.5)		12.2 (4.8)		12.9 (4.8)		8.18 (1,118)		.005
**Years of substance use**											
	Cannabis, mean (SD)	7.3 (6.1)		8.1 (6.9)		7.9 (6.7)		1.03 (1,365)		.31	
	Alcohol, mean (SD)	4.5 (6.2)		5.3 (6.5)		5.1 (6.4)		1.08 (1,332)		.30	
	Alcohol, hazardous consumption^b^, mean (SD)	1.4 (3.1)		1.8 (4.0)		1.7 (3.8)		0.66 (1,318)		.42	
	Cocaine, mean (SD)	1.1 (3.8)		0.2 (1.1)		0.4 (2.1)		11.15 (1,316)		<.001	
**Substance use in last 30 days, n (%)**											
	Cannabis	94 (100)		273 (100)		367 (100)		N/A^c^		N/A	
	Alcohol	74 (78.7)		213 (78.0)		287 (78.2)		0.26 (1)^a^		.61	
	Alcohol, hazardous consumption	35 (37.2)		99 (36.2)		134 (36.5)		0.38 (1)^a^		.54	
	Tranquilizer	8 (8.5)		16 (5.8)		24 (6.5)		0.73 (1)^a^		.39	
	Cocaine	18 (19.1)		32 (11.7)		50 (13.6)		2.73 (1)^a^		.10	
	Amphetamines	23 (24.4)		49 (17.9)		72 (19.6)		2.05 (1)^a^		.15	
	Hallucinogens	3 (3.1)		22 (8.0)		25 (6.8)		1.44 (1)^a^		.23	
	Heroin	0 (0)		0 (0)		0 (0)		N/A		N/A	
	Methadone	0 (0)		1 (0)		1 (0.2)		N/A		N/A	
	Other substances	5 (5.3)		8 (2.9)		13 (3.5)		0.79 (1)^a^		.37	

^a^A chi-square test was used where indicated.

^b^Hazardous alcohol consumption is defined as 5 or more standard drinks (50 mL of spirits, 150-200 mL of wine, or 330-450 mL of beer) per day at least 3 days a week [52].

^c^N/A: not applicable.

### Main Outcomes

Both individuals with attention deficit/hyperactivity disorder (mean difference 11.53, SD 21.87; *P*<.001) and individuals without attention deficit/hyperactivity disorder (mean difference 8.53, SD 9.4; *P*<.001) reported a significant reduction in days in the preceding month on which ≥1 joint was consumed. Likewise, a significant decrease was apparent in both the reported severity of dependence score (with: mean difference 3.57, SD 3.65, *P*<.001; without: mean difference 2.47, SD 3.39, *P*<.001) and cannabis substance use disorder score (with: mean difference 6.38, SD 5.96, *P*<.001; without: mean difference 5.33, SD 6.05, *P*<.001).

A similar pattern was evident for both anxiety (GAD score—with: mean difference 4.31, SD 4.71, *P*<.001; without: mean difference 1.84, SD 4.22, *P*<.001), and depression (CES-D score—with: mean difference 10.25, SD 10.54, *P*<.001; without: mean difference 4.39, SD 10.22, *P*<.001). However, the decrease in ASRS score was significant for the group with attention deficit/hyperactivity disorder (mean difference 4.65, SD 4.44; *P*<.001) but that for the group without attention deficit/hyperactivity disorder was not (mean difference 0.83, SD 4.10; *P*=.19).

There were no significant differences in change in use (*P*=.08), dependence (*P*=.95), use disorder (*P*=.85), attention deficit/hyperactivity disorder status (*P*=.84), depression (*P*=.84), and anxiety (*P*=.26) between baseline and final follow-up, dependent on ASRS score >14 (ie, having attention deficit/hyperactivity disorder) (Table 3 and Table 4).

Attention deficit/hyperactivity disorder symptom severity at baseline was not associated with reduced cannabis consumption (*P*=.19), severity of dependence (*P*=.14), or cannabis use disorder scores (*P*=.69); however, more severe attention deficit/hyperactivity disorder symptoms at baseline were associated with greater reductions in depression (*P*<.001) and anxiety (*P*<.001) after the intervention (Table 5).

**Table 3:** Comparison between outcomes at baseline and at the 3-month follow-up (intention-to-treat analysis)

Outcome	Without attention deficit/hyperactivity disorder (n=273)					With attention deficit/hyperactivity disorder (n=94)			
	Baseline, mean (SD)	Follow-up, mean (SD)	Effect size, Cohen *d*	95% CI	Baseline, mean (SD)		Follow-up, mean (SD)	Effect size, Cohen *d*	95% CI
Number of days cannabis (≥1 joint) was consumed in preceding 30 days	25.00 (6.40)	16.49 (10.04)	1.01	0.70 to 1.31	26.59 (4.93)		15.05 (9.76)	0.77	0.59 to 0.94
Severity of Dependence Scale	7.10 (3.06)	4.63 (3.02)	0.81	0.51 to 1.11	9.13 (3.02)		5.55 (2.86)	0.65	0.47 to 0.82
Cannabis Use Disorder Identification Test	19.59 (5.65)	14.26 (6.40)	0.88	0.58 to 1.18	23.72 (4.61)		17.34 (5.87)	0.50	0.33 to 0.67
Adult ADHD Self-Report scale	9.00 (2.95)	8.17 (4.01)	0.24	−0.05 to 0.52	15.80 (1.88)		11.15 (4.40)	0.58	0.41 to 0.75
Center for Epidemiological Studies Depression scale	19.77 (10.53)	15.38 (9.96)	0.59	0.30 to 0.88	27.41 (9.06)		17.16 (10.03)	0.75	0.57 to 0.92
Generalized Anxiety Disorder scale	6.57 (4.49)	4.71 (3.53)	0.46	0.17 to 0.75	10.68 (4.87)		6.37 (3.56)	0.76	0.59 to 0.93

**Table 4:** Regression analysis of changes in cannabis consumption, attention deficit/hyperactivity disorder, depression, and anxiety 3 months after initiating the intervention (intention-to-treat analysis, n=282)

Outcome variable	*B* (SE)	95% CI	*t* value	*P* value
Number of days cannabis (≥1 joint) was consumed in preceding 30 days	−3.72 (2.14)	−7.18 to 8.58	−1.73	.08
Severity of Dependence Scale	0.03 (0.68)	−1.31 to 1.39	0.05	.95
Cannabis Use Disorder Identification Test	0.26 (1.41)	−1.48 to 7.16	0.18	.85
Adult ADHD Self-Report scale	0.20 (1.03)	1.38 to 5.54	0.20	.84
Center for Epidemiological Studies Depression Scale	0.20 (1.03)	1.38 to 5.54	0.20	.84
Generalized Anxiety Disorder scale	0.89 (0.80)	1.24 to 3.40	1.11	.26

**Table 5:** Spearman rank correlation coefficients for baseline attention deficit/hyperactivity disorder symptom severity versus primary and secondary outcome change scores.

Outcome variable	Spearman *ρ*	95% CI	*t* value	*P* value
Number of days cannabis (≥1 joint) was consumed in preceding 30 days	0.14	−0.07 to 0.33	1.31	.19
Severity of Dependence Scale	0.11	−0.04 to 0.26	1.49	.14
Cannabis Use Disorder Identification Test	0.04	−0.16 to 0.24	0.39	.69
Center for Epidemiological Studies Depression Scale	0.33	0.19 to 0.46	4.33	<.001
Generalized Anxiety Disorder scale	0.28	−0.13 to 0.42	3.57	<.001

### Retention

**Figure 1:**
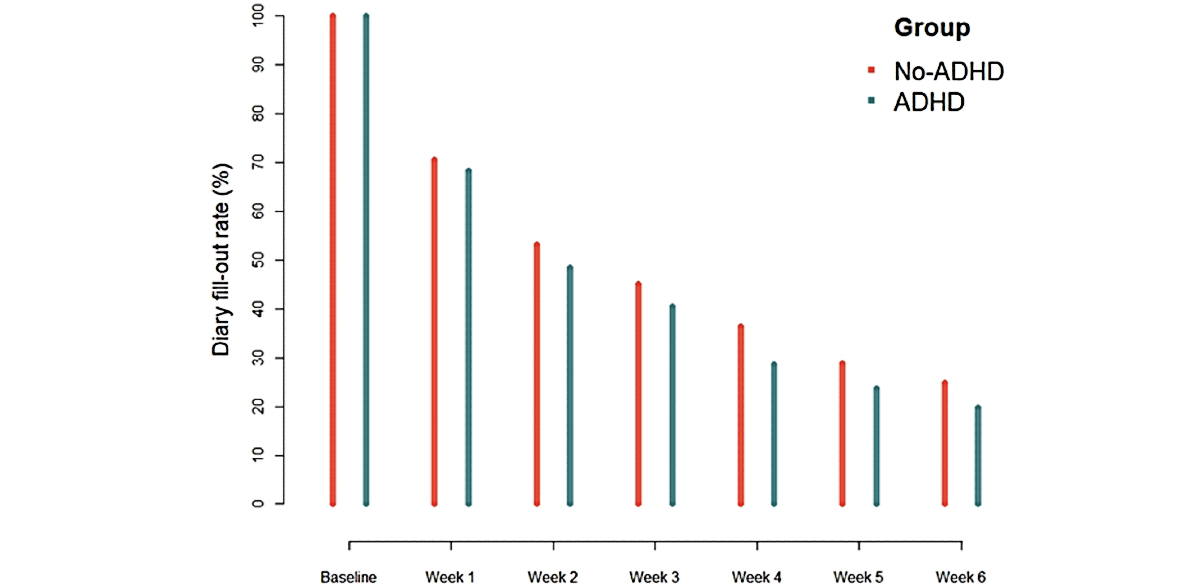
Retention throughout the study period of participants who screened positive for attention deficit/hyperactivity disorder (ADHD) (blue) versus those who screened negative (red).

Figure 1 shows the rate at which participants made entries into the consumption diary from week 1 through week 6 after baseline, and at the final follow-up assessment for both study groups. A significant intergroup difference was apparent at the final 3-month follow-up assessment, with a lower percentage of individuals having filled out the diary in the individuals with attention deficit/hyperactivity disorder than the individuals without attention deficit/hyperactivity disorder screen group (*χ*_1_^2^=5.21, *P*=.02). Overall, there was no significant association between baseline attention deficit/hyperactivity disorder symptom severity and retention rate (*ρ*=−0.10, *P*=.13).

### Participant Adherence

There was no statistically significant difference (*t*_159.27_=0.96, *P*=.34) in the number of modules completed among those screened positive for attention deficit/hyperactivity disorder (mean 2.10, SD 2.33) and those who screened negative (mean 2.36, SD 2.36). There also was no significant association between the magnitude of decrease in attention deficit/hyperactivity disorder symptoms and the number of completed modules (*ρ*=−0.09, *P*=.43).

### Safety

Of 55 individuals who completed the questionnaire on adverse intervention effects, 44 (80%) answered that they had not experienced any negative effects during the study, while 7 people (12.7%) answered that an adverse effect had affected them somewhat negatively, 3 people (5.4%) answered that an adverse effect had affected them quite negatively, and 1 person (1.8%) an adverse effect had affected them to a great extent. There was no significant difference in the percentage of individuals screening positive with attention deficit/hyperactivity disorder and individuals screening negative with attention deficit/hyperactivity disorder who reported adverse effects (*P*=.33).

## Discussion

### Principal Results

In this study, we aimed to evaluate whether the CANreduce 2.0 program can reduce cannabis use in adults who screen either positive or negative for attention deficit/hyperactivity disorder, and whether individuals screening positive might benefit more from the program than those screening negative. Furthermore, we aimed to determine whether individuals with a positive attention deficit/hyperactivity disorder screen and more severe attention deficit/hyperactivity disorder symptoms might benefit most from the CANreduce 2.0 program. The study’s main finding was that participation in the CANreduce 2.0 program reduced cannabis consumption from baseline to follow-up, both among individuals who screened positive and individuals who screened negative for attention deficit/hyperactivity disorder. Both SDS scores and CUDIT scores, indicating that the severity of dependence (both groups: *P*<.001) and frequency of cannabis use (both groups: *P*<.001), respectively, also were significantly lower after participation in the program; however, no significant differences (SDS: *P*=.14; CUDIT: *P*=.69) in the magnitude of reduction in these scores were apparent between participants who screened positive for attention deficit/hyperactivity disorder and those who screened negative. Similarly, psychological comorbidities—such as anxiety and depression—also improved after program participation, with significant changes observed in both the attention deficit/hyperactivity disorder positive and negative screening groups (*P*<.001). Participants with more severe attention deficit/hyperactivity disorder symptoms at baseline exhibited a greater reduction in depression and anxiety than those with milder symptoms. The rate of retention was significantly less (*P*=.02) in the individuals with attention deficit/hyperactivity disorder group at the end of the study period 3 months after starting the program, while attention deficit/hyperactivity disorder symptom severity was not significantly associated with the retention rate (*P*=.13). Those screening positive for attention deficit/hyperactivity disorder and those screening negative did not significantly differ in their adherence to the program (*P*=.43) or the number reporting adverse events (*P*=.33).

Although cannabis is a widely used psychoactive substance that may induce dependency and cause associated problems, many users do not seek help at outpatient addiction centers. Reasons for this include the relative inaccessibility of treatment centers, fear of stigmatization, and inadequate awareness that treatment is needed [40]. Web-based self-help programs have yielded beneficial results for alcohol and tobacco users [53-56], but information on the efficacy of such programs for cannabis users is limited. Users with comorbid psychiatric diseases, such as attention deficit/hyperactivity disorder, might particularly benefit from web-based self-help with adherence-focused guidance, as their attention deficit/hyperactivity disorder could innately aggravate their ability to adhere to a program lacking such support. However, no significant differences between the 2 screening groups in our study (those screening positive vs negative for attention deficit/hyperactivity disorder) were evident, in terms of reducing cannabis consumption (*P*=.08), the severity of dependence (*P*=.95), cannabis substance use disorder (*P*=.85), anxiety (*P*=.26), or symptoms of depression (*P*=.84), as all these outcomes were reduced similarly in the 2 groups. Hence, the program appears to provide similar benefits to cannabis users who screen positive and those who screen negative for attention deficit/hyperactivity disorder, which is contrary to our expectation of a particular benefit for users with attention deficit/hyperactivity disorder.

Participants who screened positive for attention deficit/hyperactivity disorder filled out the consumption diary to a lesser extent over the course of follow-up. It is, therefore, possible that participants screening positive for attention deficit/hyperactivity disorder used the intervention less than those without attention deficit/hyperactivity disorder over time and, as such, the program’s effects might have been blunted in that group. At the same time, there were no differences in adherence between the 2 groups, which means that, over the course of the intervention, those screening positive and negative for attention deficit/hyperactivity disorder participated in the program to roughly the same degree. This is an encouraging outcome, as it potentially indicates that individuals with attention deficit/hyperactivity disorder can, indeed, participate in and adhere to a web-based remote intervention such as CANreduce 2.0. However, it was our assumption that those with more attention deficit/hyperactivity disorder symptoms would profit most from adherence-focused guidance. This assumption of ours was based on the beneficial effect of guidance in internet-based interventions [57] and the known deficits of individuals with attention deficit/hyperactivity disorder regarding impulse inhibition, working memory, organization, and planning skills [58].

Unfortunately, no conclusion can be drawn regarding possible withdrawal symptoms in our study; we did not assess withdrawal symptoms because we had previously found [50] that the vast majority of persons reduced their cannabis use slowly and gradually, and probably with no or only occasionally very mild withdrawal symptoms occurred.

In our study sample, we observed differences in the completion rates for individual modules in the CANreduce 2.0 program between participants who screened positive versus negative for attention deficit/hyperactivity disorder. Baseline differences between these groups in their CUDIT, ASRS, SDS, CES-D, and GAD-7 scores might explain these differences, given that individuals screening positive for attention deficit/hyperactivity disorder entered the program with higher scores for all these measures.

Overall, the only significant difference between those with attention deficit/hyperactivity disorder and those without attention deficit/hyperactivity disorder was the reduction in mean attention deficit/hyperactivity disorder score between baseline and final follow-up (*P*<.001); however, individuals with attention deficit/hyperactivity disorder had higher initial attention deficit/hyperactivity disorder severity scores, which could explain this difference in the degree of improvement. That the severity of attention deficit/hyperactivity disorder-specific symptoms was reportedly reduced after the intervention is highly relevant, because it indicates that a cannabis-specific intervention can lead to reduced attention deficit/hyperactivity disorder severity. However, this might be a cannabis dose–dependent and not a simple direct effect, and verifying this would require controlled pharmacological research. That attention deficit/hyperactivity disorder severity at baseline was inversely correlated with the extent of change in comorbid anxiety and depression indicated that potential effects of the program might have been overlooked, as a result of the allocation of participants based on predefined threshold of symptoms.

The mean number of completed modules was very low for both, individuals who screened positive for attention deficit/hyperactivity disorder severity (mean 2.10, SD 2.33) and individuals who screened negative for attention deficit/hyperactivity disorder severity (mean 2.36, SD 2.36). This number is low because approximately one-third of participants only logged in briefly, worked through the first module, and never logged in again. These early dropouts are a common problem in web-based interventions [59], especially with interventions that set the hurdles for study participation low (exclusively web-based recruitment, broad inclusion criteria, and only a web-based baseline questionnaire).

On the other hand, a sizeable proportion of participants stayed in the program for the first 2 weeks and then worked through a large number of the modules. Nevertheless, efforts should continue to find ways to reduce the high dropout rates experienced with this web-based program. The next steps to improve the program could be, for example, identifying through qualitative interviews the background and evaluations of the program by participants screening positive versus those screening negative for attention deficit/hyperactivity disorder.

### Limitations

This study has certain limitations that must be considered. First, there were baseline differences between the 2 study groups, including a higher number of cannabis use days in those screening positive for attention deficit/hyperactivity disorder. Second, the follow-up duration of 3 months might have been too short to establish any long-term effectiveness of the CANreduce 2.0 program; a longer period of follow-up could provide insights into how well program users retain whatever benefits they appear to achieve from the program. Third, the influence of personalized counseling on the observed outcome, being just one component of the program, cannot be ascertained. Fourth, formally diagnosing attention deficit/hyperactivity disorder requires at least one detailed face-to-face assessment; but given that this study targeted individuals who overuse cannabis in the general population who were not otherwise in treatment for their cannabis use, we had no choice but to rely on a web-based attention deficit/hyperactivity disorder screening instrument and no actual person-to-person contact. Fifth, potential, unrecognized confounders might have resulted from individuals not being randomly assigned to the 2 study groups of interest—those screening positive versus negative for attention deficit/hyperactivity disorder—and any one of these potential confounders could have biased results. Sixth, our analyses were limited by a sample size that was powered for the main study and not specifically for comparisons of those screening positive versus negative for attention deficit/hyperactivity disorder. Seventh, 2 items in the ASRS screening tool, which we used to measure attention deficit/hyperactivity disorder symptoms, might be prone to elevated scores in heavy cannabis users who do not suffer from attention deficit/hyperactivity disorder. False-positive results could, thereby, result from potential overlap of symptoms between attention deficit/hyperactivity disorder and cannabis use. Specifically, this refers to the items measuring appointment or obligation forgetfulness and task avoidance or delay. Furthermore, our decision to apply the ASRS [60] to measure attention deficit/hyperactivity disorder symptoms was based on our need to keep the assessment time brief. However, more extensive scales exist such as the KATE [61] or CAARS [62] would have most likely provided more reliable results.

### Conclusions

The CANreduce 2.0 program appeared to benefit both individuals with and without attention deficit/hyperactivity disorder similarly. This web-based program offers a personalized counselling option designed to increase intervention adherence and may provide a viable option to reach cannabis users remotely, including those with attention deficit/hyperactivity disorder, and improve both psychiatric comorbidities and overall condition.

## Appendix

Multimedia Appendix 1 CONSORT-eHEALTH checklist (V 1.6.1).

## Abbreviations

ASRS: Adult ADHD Self-Report Scale
CES-D: Center for Epidemiologic Studies Depression scale
CUDIT: Cannabis Use Disorder Identification Test
GAD: Generalized Anxiety Disorder scale
SDS: Severity of Dependence Scale
